# Anisotropy of 3D Columnar Coatings in Mid-Infrared Spectral Range

**DOI:** 10.3390/nano11123247

**Published:** 2021-11-29

**Authors:** Lina Grineviciute, Soon Hock Ng, Molong Han, Tania Moein, Vijayakumar Anand, Tomas Katkus, Meguya Ryu, Junko Morikawa, Mark J. Tobin, Jitraporn Vongsvivut, Tomas Tolenis, Saulius Juodkazis

**Affiliations:** 1 Center for Physical Sciences and Technology, Savanoriu̧ Ave. 231, LT-02300 Vilnius, Lithuania; lina.grineviciute@ftmc.lt; 2 Optical Sciences Centre and ARC Training Centre in Surface Engineering for Advanced Materials (SEAM), School of Science, Swinburne University of Technology, Hawthorn, VIC 3122, Australia; soonhockng@swin.edu.au (S.H.N.); molonghan@swin.edu.au (M.H.); tmoein@swin.edu.au (T.M.); vanand@swin.edu.au (V.A.); tkatkus@swin.edu.au (T.K.); 3 Melbourne Centre for Nanofabrication (MCN), Australian National Fabrication Facility, Clayton, VIC 3168, Australia; 4 Research Institute for Material and Chemical Measurement, National Metrology Institute of Japan (AIST), Tsukuba Central 3, 1-1-1 Umezono, Tsukuba 305-8563, Japan; ryu.meguya@aist.go.jp; 5 CREST—JST and School of Materials and Chemical Technology, Tokyo Institute of Technology, 2-12-1, Ookayama, Meguro-ku, Tokyo 152-8550, Japan; morikawa.j.aa@m.titech.ac.jp; 6 World Research Hub Initiative (WRHI), School of Materials and Chemical Technology, Tokyo Institute of Technology, 2-12-1, Ookayama, Meguro-ku, Tokyo 152-8550, Japan; 7 Infrared Microspectroscopy (IRM) Beamline, ANSTO-Australian Synchrotron, 800 Blackburn Road, Clayton, VIC 3168, Australia; tobinm@ansto.gov.au (M.J.T.); jitrapov@ansto.gov.au (J.V.); 8 ELI Beamlines, Institute of Physics, The Czech Academy of Sciences, Za Radnicí 835, 252 41 Dolní Břežany, Czech Republic

**Keywords:** polarisation, IR, fingerprint region, birefringence, dichroism, sculptured thin films, anisotropy

## Abstract

Polarisation analysis in the mid-infrared fingerprint region was carried out on thin (∼1 μm) Si and SiO_2_ films evaporated via glancing angle deposition (GLAD) method at $\;70^{\circ }$ to the normal. Synchrotron-based infrared microspectroscopic measurements were carried out on the Infrared Microspectroscopy (IRM) beamline at Australian Synchrotron. Specific absorption bands, particularly Si-O-Si stretching vibration, was found to follow the angular dependence of ∼$\cos^{2}\theta$, consistent with the absorption anisotropy. This unexpected anisotropy stems from the enhanced absorption in nano-crevices, which have orientation following the $\cos^{2}\theta$ angular dependence as revealed by Fourier transforming the image of the surface of 3D columnar films and numerical modeling of light field enhancement by sub-wavelength nano-crevices.

## 1. Introduction

Glancing angle deposition (GLAD) [1] can be used to make highly anisotropic thin films for light polarisation control, namely waveplates suited for the linear and circular polarisations [2,3] and polarisers for zero angle of incidence [4], by utilising complex arrangements of sample’s tilt and rotation [5]. It becomes possible to make strongly birefringent thin films by GLAD using optically isotropic Si and SiO_2_ materials [6]. It was recently demonstrated that such films have larger visible and near-infrared (near-IR) laser damage thresholds in J/cm${}^{2}$ compared to polished surfaces or coatings made out of the two different materials [7]. GLAD deposited thin films can also act as an anti-reflection coating [8,9]. Recently, numerous investigations have been reported regarding the simulations and analysis of the nanostructure of the GLAD coatings [10,11] and constant morphology changes during growth [12] or in time [13]. Both silicon and silica have been explored for control of birefringence and structural properties, including porosity and stoichiometry [14], using GLAD. Anisotropic Si has shown birefringence up to 0.25 in the IR spectral range (∼1500 nm) [15], while it was lower for silica; however, an augmented optical damage threshold was found in the case of silica in the UV-VIS range [3]. In both cases, the refractive index anisotropy of a film was created from an amorphous (isotropic) material, which can be applied on virtually any surface, including glass substrates [4] or laser crystals [16]. Such films are promising for development of micro-laser systems.

All results indicate that deeper understanding is required in order to fully control the properties of nanostructured thin films, especially when stoichiometry changes [14]. How such anisotropic films behave at longer wavelengths in mid-IR fingerprint region needs clarification for applications in high-intensity laser optics, as well as in surface enhanced Raman scattering (SERS) sensors [17]. In addition, the birefringent films and optical elements made by GLAD are porous and possess high surface area. The key question on whether surface defect absorption can compromise the performance of such GLAD coatings due to increased absorption deserves deeper analysis.

The mid-IR spectral range of 400–4000 cm${}^{-1}$ is indispensable for material characterisation. In this study, we used the IR radiation produced by a synchrotron source that offers 100–1000 times higher brightness than that of a thermal IR source commonly used in a laboratory-based FTIR instrument. This highly intense and highly collimated synchrotron-IR beam enhances spectral quality, in terms of signal-to-noise (S/N) ratio, and enables acquisition of high-quality FTIR spectra at diffraction-limited spatial resolution ideal for analysis of these micro-films.

We showed previously [18] that separate contributions to anisotropy due to birefringence and dichroism related to the real and imaginary parts of the refractive index (n+iκ), respectively, can be revealed from the angular dependence of transmittance $T=\frac{I_{T}}{I_{0}}$ where the transmitted and incident light intensities are $I_{T}$ and $I_{0}$, respectively. The absorbance, i.e., the optical density A≡OD=−lgT (for a negligible reflectance *R*). Indeed, an absorbing dipole has minimum and maximum *A* (and *T*) within an orientation change of π. Transmitted power through a linear polariser (e.g., mesh grid absorber) is given by the Malus law $T\propto \cos^{2}\theta$. However, a change of *T* due to birefringence doubles the angular frequency since min-max of *T* occurs twice within an orientation change of π. The angular dependence of transmittance T(θ), when reflectance and absorbance are negligible R→0, A→0 for the crossed (×) polariser–analyser setup, is given by:

(1) $$T_{\times }\left(\theta \right)=\sin^{2}2\left(\theta -\theta_{R}\right)\sin^{2}\left(\pi \Delta nd/\lambda \right),$$

where θ is the orientation angle; $\theta_{R}$ is the slow or fast axis direction (i.e., the slow axis is usually aligned to the main molecular chain or along a polymer stretch direction); Δn is the birefringence of the sample/object at the wavelength λ, for thickness *d*. Hence, $T\propto \sin^{2}\left(2\theta \right)$ angular dependence, which is double the angular frequency 2θ, rather θ for the absorber (Malus law). Following energy conservation, the absorptance Abs=1−R−T, the optical density OD is defined by $T=\left(1-R\right)\times 10^{-OD}$, where *R* and *T* can be directly measured and $R=\frac{I_{R}}{I_{0}}$ with $I_{R}$ reflected intensity; accordingly, $Abs=\left(1-R\right)\times \left(1-10^{-OD}\right)$.

Here, we reveal optical absorption anisotropy in the mid-IR fingerprinting region of 3D columnar films deposited by GLAD using the polarised synchrotron-IR microspectroscopy. Structural characterisation by scanning electron microscopy (SEM) was used and numerical modeling was applied for qualitative analysis.

## 2. Results

**Figure 1:**
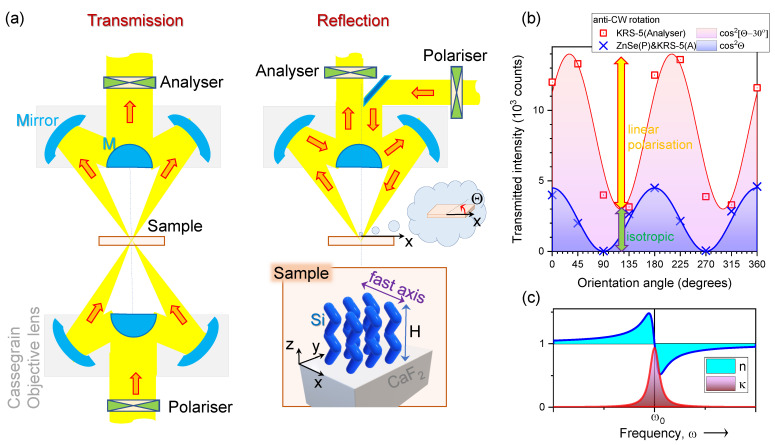
(**a**) Transmission and reflection modes with the possibility to set polarisation of the incident IR beam and analysis at the transmitted (or reflected) light; θ is sample’s orientation angle with $\theta =0^{\circ }$ corresponding to the *x*-axis. Samples were Si and SiO${}_{2}$ columnar structures of H≈1μm height deposited by GLAD at $\phi ∼70^{\circ }$ on a substrate (silica, CaF${}_{2}$, Si). The sketch shows Si columnar structures deposited via zig-zag folding (6 s deposition switch) along the *x*-axis during GLAD on IR transparent CaF${}_{2}$; folding direction defines the optical fast-axis with refractive index $n_{e}<n_{o}$ due to structural anisotropy; (**b**) transmitted power through one (red) and two (blue) crossed polarisers used in experiments. A crossed polariser (P)—analyser (A) eliminated transmitted power; measurements were carried out in transmisssion mode with a 5.6 μm aperture pinhole, which was contributing to a better axial resolution; (**c**) Lorenzian lineshapes for absorption $A\left(\omega \right)=\frac{\tau }{1+{\left(\omega -\omega_{0}\right)}^{2}\tau^{2}}$ and dispersion $1D\left(\omega \right)=1-\frac{\left(\omega -\omega_{0}\right)\tau^{2}}{1+{\left(\omega -\omega_{0}\right)}^{2}\tau^{2}}$, where ω is the cyclic frequency and τ is the relaxation time [19]; κ∝A(ω) and n∝D(ω) where refractive index is $\sqrt{\varepsilon }=\left(n+i\kappa \right)$.

Polarisation analysis of thin columnar films deposited by GLAD was carried out in transmission *T* and reflection *R* modes (Figure 1a) depending on transparency of the substrate in the 600–4000 cm${}^{-1}$ spectral window (16.7–2.5 μm in wavelength). To ensure that the region on the substrate from which the orientational dependence of transmittance T(θ) and/or reflectance R(θ) was measured is consistent, wire grid KRS-5 (thallium bromo-iodide) and ZnSe polarisers were rotated with a sample at a fixed position. The orientation angle θ corresponds to the angle between polariser and sample orientations. This ensured measurement from the very same focal spot, typically ∼17 μm in diameter (on the sample). Figure 1b shows one polariser and crossed polariser–analyser transmission intensity. Due to the linear and isotropic (circularly polarised) contributions of the dipole and edge emission from the synchrotron source, it was not possible to extinguish transmittance with one polariser. With a pair of crossed polariser and analyser, it was possible to reach near-zero transmittance.

The degree of linear polarisation from synchrotron radiation [20,21] reaching the beamline is defined by the powers of linear $P_{L}$ and isotropic $P_{I}$ components, respectively, as $Lin=P_{L}/\left(P_{L}+P_{I}\right)=11/14\approx 78.6\%$ (Figure 1b). This is consistent with the 22% previously measured for linearly polarised radiation at the THz beamline, which uses the other half of the synchrotron-IR beam extracted by the first IR mirror, with a larger portion of the isotropic emission [22]. With the strong linearly polarised synchrotron radiation at the Infrared Microspectroscopy (IRM) beamline, polarisation dependence of absorbance and transmittance can be analysed at an enhanced S/N ratio. Orientation of the slit of the first IR mirror defines the orientation of a linearly polarised component, which was along the *x*-axis in our experiments.

Polarisation analysis in the mid-IR spectral range can be carried out with the described polariser–analyser setup in direct transmission as well as in attenuated total reflection (ATR) modes [23]. Less explored polarisation dependencies are at the vicinity of the absorbance bands where the Lorenzian absorption A(ω) and dispersion D(ω) lineshapes are interrelated [19] (Figure 1c).

### 2.1. Polarised Synchrotron-IR Spectra of Anisotropic Columnar Si Micro-Films

**Figure 2:**
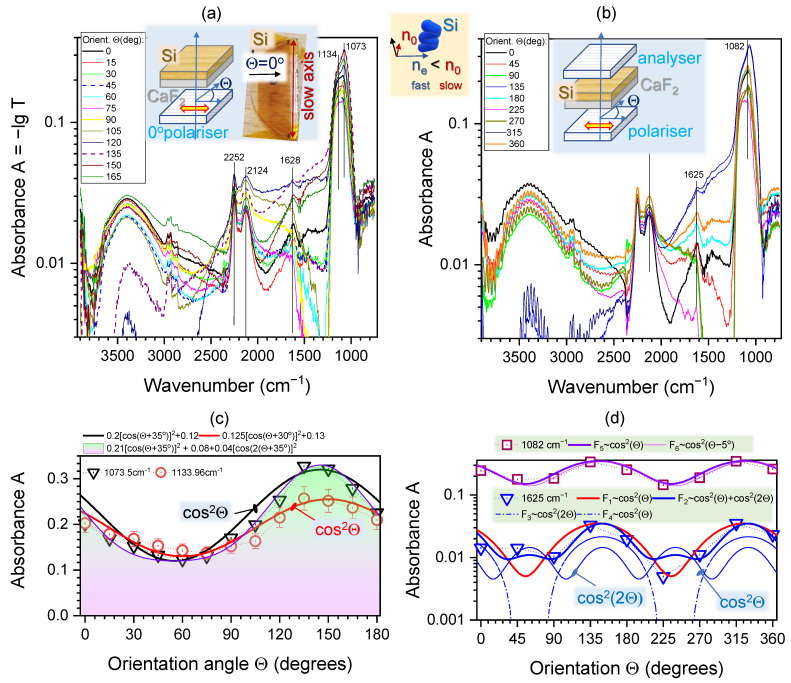
Polarisation analysis of the absorption bands. (**a**) Spectra at various polarisation orientations of θ for an analyser only setup. Inset shows geometry of experiment and a top-view photo of the sample with $\theta =0^{\circ }$ along the fast-axis ($n_{e}$ index); slow-axis ($n_{o}$) is at $\theta =90^{\circ }$. Positive θ corresponds to the anti-clockwise rotation around the *z*-axis. (**b**) spectra at various orientations of θ polarisation for an aligned polariser–analyser setup; (**c**) orientational dependence of absorbance A(θ) of a columnar Si of H≈1μm height measured at the polarised irradiance; reference was CaF${}_{2}$ substrate. Error bars mark ±10% uncertainty band; (**d**) the dependence A(θ) and spectra measured through an aligned polariser–analyser pair (high transmission mode). Focal spot size 16.7 μm; Polariser is KRS-5 and Analyser ZnSe (see insets and Figure 1); spectral resolution 4 cm${}^{-1}$. See details in the text. Note the lg-scale.

Figure 2a shows polarised synchrotron-IR spectra of 3D columnar Si deposited on an IR-transparent CaF_2_ substrate. A single polariser (analyser) was rotated through $180^{\circ }$ in $15^{\circ }$ intervals and changes in spectral features are shown for each orientation θ. Figure 2b shows the same sample, but measured with an aligned polariser–analyser setup. The strongest absorption was found at ∼1100 cm${}^{-1}$ attributable to Si-O-Si stretching vibration [24]. A Si-C band at ∼1620 cm${}^{-1}$ was observed close to the adventitious carbon band at ∼1640 cm${}^{-1}$ (i.e., C=C stretching mode) [25]. The doublet peaks centred at 2250 and 2120 cm${}^{-1}$ are assignable to Si-H and Si-H_2_ vibrations, respectively [26]. Their positions are located adjacent to the CO_2_ peaks (∼2330 cm${}^{-1}$) [27], which are recognisable as an atmospheric interference (the reference sample used for background measurement in this study was CaF_2_). Due to inherent porosity of 3D columnar Si film, a perfect cancellation of the gaseous CO_2_ bands was not achieved, even though samples were held in a nitrogen-purged enclosure throughout the experiment. Distinct CH_3_ and CH_2_ stretching bands in the 2950–2850 cm${}^{-1}$ spectral region are identifiable and observed next to the broad -OH stretching band at ∼3400 cm${}^{-1}$ as a result of the Si-OH structure. Interestingly, there is a similarity of a spectral profile of absorbance between 3D porous columnar Si film deposited by GLAD and 3D porous Si made by electrochemical wet etching of (poly)crystalline Si [28].

We selected several spectral positions within specific absorption bands of Si-O-Si and some other spectrally distinct locations to investigate anisotropy of birefringence and dichroism at the corresponding wavelengths.

### 2.2. Orientation Dependence of IR Absorption Bands

In order to measure polarisation dependence of absorbance A∝Δκ, one polariser is required. For measurement of birefringence $\Delta n=n_{e}-n_{o}$, usually a cross-polarisation setup is used and transmittance $T_{\times }$ (Equation (1)) is measured. However, such determination of birefringence is not applicable to absorbing samples or at spectral positions close to absorption bands. In the visible spectral range, where most polarisation optical elements are used (T→1), absorption is not important and optical losses are mostly due to reflection and scattering (related to Δn rather Δκ). It is possible, however, to determine the dichroism Δκ contribution in the case of polariser–analyser transmission measurement when they are both aligned to the maximum transmittance (‖). This condition is related to the cross-polarisation alignment by simple algebraic expression $T_{\|}=1-T_{\times }$ (see Equation (1)). Indeed, for determination of optical losses due to absorption (and its anisotropy Δκ), a high transmission measurement setup is required in contrast to the measurement of birefringence Δn where crossed polarisation (zero transmission) is implemented.

It was shown that separation of anisotropy due to birefringence Δn and dichroism Δκ can be obtained using a fit to the transmittance T(θ) (or absorbance A(θ)=−logT(θ)) by the cos(2θ) and cosθ, respectively [18]. Such analysis is presented in Figure 2c,d. Different fits are shown for qualitative understanding of anisotropy components measured with analyser and aligned polariser–analyser (hence $T_{\|}$).

**Figure 3:**
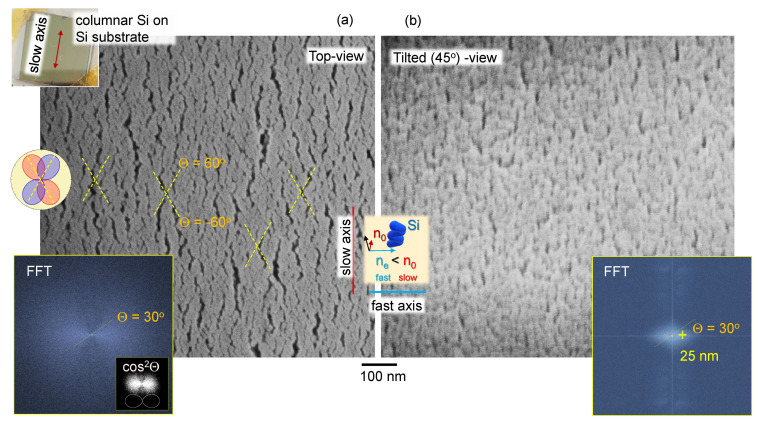
Surface morphology of columnar Si film on Si substrate. (**a**) SEM images of a ∼1 μm-thick columnar Si (top-left inset photo of 1×1cm${}^{2}$ sample); no metal coating was used for SEM imaging; side-insets show fast Fourier transform (FFT) images of top and slanted view images; (**b**) high-contrast FFT image (top-view SEM) shows an overlaid $\cos^{2}\theta$ polar plot. Center-inset shows orientation of the columnar Si coating. The cross-markers (dashed lines) on the top-view image show orientations of crevices at $\pm 60^{\circ }$ orientations and corresponding dipole profiles $\cos^{2}\left(\theta \pm 60^{\circ }\right)$.

With only the analyser, one could reveal anisotropy in absorbance if there is ordering and alignment of the absorbing dipoles. However, if the sample is isotropic, there should be no angular dependence in A(θ). For example, the absorption band, e.g., Si-O-Si at ∼1100 cm${}^{-1}$ should follow $T\left(\theta \right)\propto \cos^{2}\theta$ in the presence of the simplest case of anisotropy. Experimental data of A(θ) were fitted and the orientation angle was $\theta_{D}=35^{\circ }$: $\cos^{2}\left(\theta +\theta_{D}\right)$. It was different from the orientation of the transmission maximum shown in Figure 1b by ∼65${}^{\circ }$. The sample of columnar (normal to the substrate) Si film has a form–birefringence at visible wavelengths, i.e., there is an alignment due to a specific material distribution with an extraordinary refractive index smaller than the ordinary $n_{e}<n_{o}$. How such a structure can affect the anisotropy property in mid-IR region has never been reported and requires further investigation. Structure of a pristine surface of columnar Si film without metal coating is shown in Figure 3. This film was deposited on a Si substrate (not CaF${}_{2}$) to eliminate surface charging during SEM imaging and the as-fabricated structure of columnar Si film can be visualised.

The $\cos^{2}\theta$ fits of columnar Si on CaF${}_{2}$ (see detailed expressions in the legend of Figure 2c) closely match A(θ). However, a slightly better fit can be achieved with two contributions. Namely, for the 1073 cm${}^{-1}$ experimental measurements of A(θ) with $\Delta \theta =15^{\circ }$ steps, the fit as a sum of θ (dichroism) and 2θ (birefringence) is better (Figure 2c). This terminology of θ and 2θ dependencies are used here for brevity. It should be remembered that the transmittance *T* (so also absorbance *A*) follows $T\propto \cos^{2}\theta$ the θ-dependence (folding onto itself with cycle of π). However, for the *A*, fits should not be confused with apparent 2θ dependence: $\cos^{2}\theta =\frac{1}{2}\left(\cos\left(2\theta \right)+1\right)$. This hints that 3D form-birefringent Si films (Figure 3) can have a contribution to the pure absorption band (atomic/molecular level dipole alignment) due to a larger microscale material alignment and reorganisation.

### 2.3. Transmittance through an Aligned Polariser–Analyser Setup $T_{\|}$

Fit of experimentally measured absorbance A(θ)=−logT in the aligned polariser–analyser setup can separate contributions due to the birefringence and dichroism when the following fit is used [18]:

(2) $$A_{\|}\left(\theta \right)=\left[Amp_{D}\times \cos^{2}\left(\theta -\theta_{D}\right)+{\mathrm{Offset}}_{D}\right]+\left[Amp_{R}\times \cos^{2}2\left(\theta -\theta_{R}\right)+{\mathrm{Offset}}_{R}\right],$$

where *D* index stands for contributions due to absorption dichroism and *R* is due to birefringence (real part of the refractive index). In order to check the above presented conjecture that birefringence contribution to the absorption band should be considered due to sub-wavelength nano-structure of the 3D Si film, measurements were carried out with an aligned polariser–analyser setup by measuring $A_{\|}\left(\theta \right)=-\mathrm{lg}T_{\|}\left(\theta \right)$ shown in Figure 2b. This measurement is suitable to separate θ and 2θ dependencies using fit (Equation (2)). Figure 2b shows that the strongest Si-O-Si band has the major contribution due to the component of the θ-dependence, which was the main contribution using the analyser setup (a). The $\cos^{2}\left(2\theta \right)$ contribution in one-polariser *A* measurement might be caused by the double action of the sample which has two high order structures: the form birefringent SiO_2_ zig-zag structure and oriented nano crevices. One may act as a polariser and another as a retarder, so that one can observe two angular dependencies $\cos^{2}\left(\theta \right)$ and $\cos^{2}\left(2\theta \right)$ even under the single Nicole configuration.

Large orientation steps of Δθ=π/4 does not allow for a reliable fit by $\cos^{2}\left(2\theta \right)$. The $\cos^{2}\theta$-fits are reliable as demonstrated by a small $\Delta \theta_{R}=5^{\circ }$ change in $F_{5}$ and $F_{6}$ functions (Figure 2d). Sampling at smaller angular steps is planned for future beamtime experiments at the Australian Synchrotron IRM beamline.

A shoulder band at 1625 cm${}^{-1}$ (Figure 2d) can be fitted by Equation (2) with a weak $\cos^{2}\left(2\theta \right)$ contribution, i.e., some birefringence orientational behaviour. For qualitative analysis, it is instructive to show separate $\cos^{2}\theta$ and $\cos^{2}\left(2\theta \right)$ contributions (D and R terms in Equation (2)) to the fit. Asymmetric distribution of A(θ) can be fitted by adding π/2-shifted angular dependencies ($F_{4}$ and $F_{3}$ in Figure 2d) using the orientation angles $\theta_{R}$ and $\theta_{D}$ (with selected amplitude and offset values). The fits presented in (d) are qualitative only since a higher angular sampling is required to clearly distinguish the Δn-related high angular frequency contribution.

**Figure A1:**
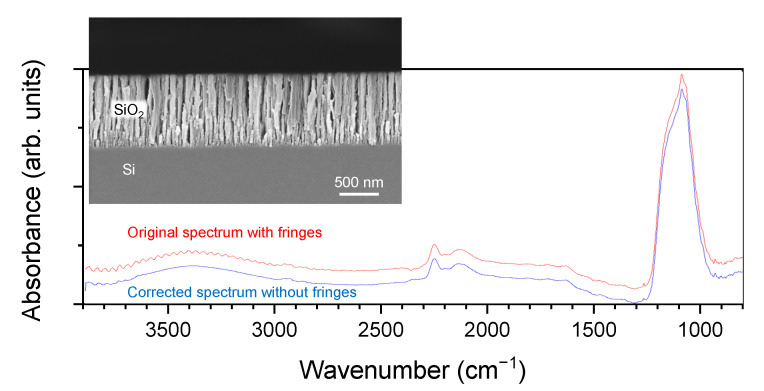
Fringe removal from Si-on-CaF${}_{2}$ spectra by the method presented in Ref. [29]; plotted with vertical offset for clarity. The inset shows a cross-sectional SEM view of SiO${}_{2}$-on-Si prepared in the same conditions; a better quality cleaving of the sample can be achieved on the Si substrate; the sample was cleaved along the fast-axis.

The broad Si-OH band at 3400 cm${}^{-1}$ shows interference features characteristic to measurement with two polarisers (mesh grids on KRS-5 and ZnSe). Those interference fringes can be filtered out by editing the Fourier interferograms, if required [29] (Figure A1 shows the corrected spectrum after performing the fringe removal). For an isotropic homogeneous sample, there should be no orientational dependence due to adsorbed (and absorbed) water in this spectral range.

**Figure 4:**
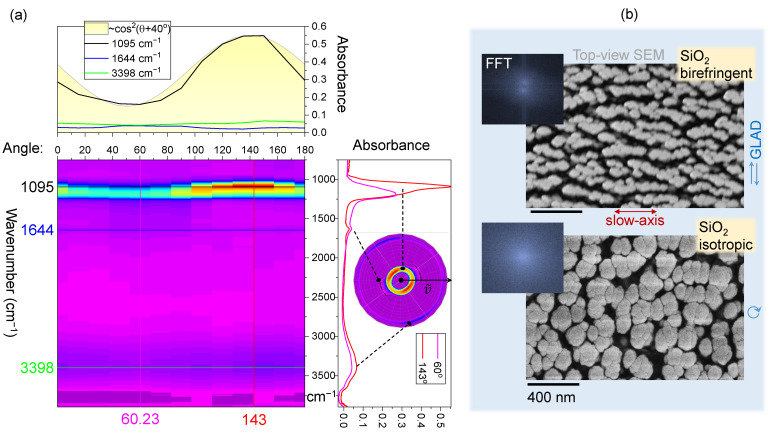
(**a**) The IR absorbance map of columnar SiO_2_ on CaF_2_ substrate with side-view cross sections of spectra at specific orientation angle θ, measured with the aligned polariser–analyser rotated around the sample. The ∼$\cos^{2}\theta$ fit is the best match to the angular dependence of the Si-O-Si band at $\tilde{\nu }=1095$ cm${}^{-1}$. The inset in the absorbance spectrum cross section shows a 2π-folded angular dependence map to highlight angular correlations between bands; (**b**) top-view SEM images of a birefringent SiO_2_ columnar film (researched in another study and coated at 74${}^{\circ }$ angle) and isotropic SiO_2_ grown by GLAD at a constant rotation; see differences in FFT maps.

Next, anisotropic SiO_2_-on-CaF_2_ samples of the same height and GLAD deposition conditions as Si-on-CaF_2_ are summarised in Figure 4a. The chemical map of selected cross sections based on the absorption intensity of the Si-O-Si band at 1095 cm${}^{-1}$ shows almost an identical angular dependence of $\cos^{2}\left(\theta +40^{\circ }\right)$ as was observed for Si-on-CaF_2_ (Figure 2). Such a dependence is attributable to the nano-crack/planes of SiO_2_ columns alignment along the optical slow-axis (for visible wavelengths). Fourier transform of SEM images shows the pattern anisotropy following the ∼$\cos^{2}\theta$ angular dependence, which is absent in the case of the same SiO_2_ columnar film grown at a constant spinning during GLAD deposition. Other peaks at 1644 and ∼3400 cm${}^{-1}$ showed almost no angular dependence. The band at 1644 cm${}^{-1}$ shows only a weak angular dependence, but has anti-correlation character with the Si-O-Si absorption at 1095 cm${}^{-1}$. The 1645 cm${}^{-1}$ band was determined from an isotropic absorption to be a combination of the phonons at 1160 cm${}^{-1}$ and at 480 cm${}^{-1}$ [30]. Interestingly, the 811 cm${}^{-1}$ phonon band was not observed in 3D columnar silica, which also contributes to a significant 2000 cm${}^{-1}$ absorption band of SiO_2_ [30]. A weak shoulder feature at 3740 cm${}^{-1}$ is attributed to Si-OH stretching modes, while the related broad band at 3660 cm${}^{-1}$ is not prominent.

## 3. Discussion

An unexpected result is that Si film, which is expected to show absorption anisotropy due to its vertical columnar (isotropic) structure, in fact, shows an angular dependence of the strongest Si-O-Si absorption band following $A\left(\theta \right)\propto \cos^{2}\left(\theta -\theta_{D}\right)$ (measured in transmission; Figure 1b). This measurement was carried out with a parallel polariser–analyser setup and was sensitive to the birefringence contribution, if any. However, Δn contribution with $\cos^{2}\left(2\theta \right)$ angular dependence was found to be negligible (finer angular sampling is required for definitive separation of θ and 2θ dependencies). The angle for the strongest absorption was $\theta_{D}\approx 65^{\circ }$ from the orientation when strongest linearly polarised synchrotron-IR radiation was focused onto the sample. Figure 3 reveals that the FFT image of top-view Si columnar coating has $\cos^{2}\theta$ distribution; for an isotropic sample, a disk or ring pattern is present (see Figure 4b). The orientation of ∼60${}^{\circ }$ corresponded to some of the largest crevices, which were formed at an angle in the direction of a slow-axis (Figure 3).

**Figure 5:**
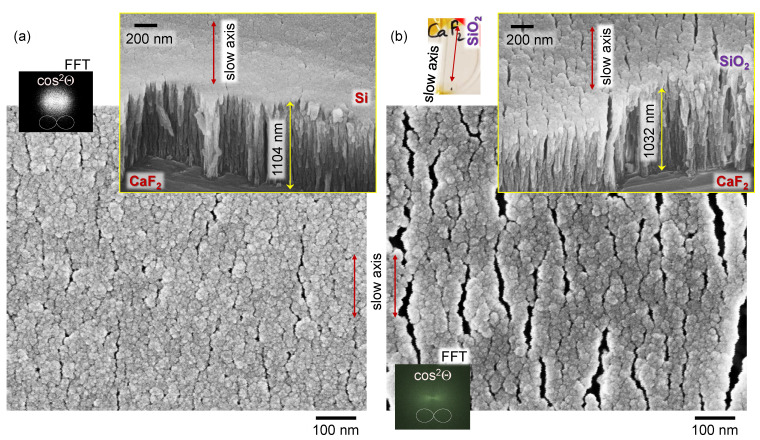
SEM images of a ∼1 μm thick columnar Si (**a**) and SiO_2_ (**b**) on CaF_2_ substrate. A coating of 20 nm thick Cr was used for SEM imaging (columnar Si film without Cr coating is shown in Figure 3). Top-insets were taken at π/4 tilt and film thickness was calculated with a factor of sin(π/4) as $H=y/\sqrt{2}$; where *y* is the vertical length on the image.

These sub-wavelength structures (at IR wavelengths of incident light) will contribute to light field enhancement and hence contribute to a stronger absorbance. This is an underlying reason why the structure/orientation of the surface features with angular dependence $\cos^{2}\theta$ is transferred to the angular dependence of absorbance A(θ). Even for isotropic columnar Si films, the angular dependence of *A* is present due to a nanoscale structure of Si film. Figure 5 shows a 3D structure of Si and SiO_2_ columnar films. A very similar structure of films was obtained at the same protocol of GLAD deposition. Slightly larger nano-crevices were observed on dielectric SiO_2_ surface, which has stronger charging under SEM imaging even when coated with 20 nm thick Cr layer for imaging purposes.

**Figure 6:**
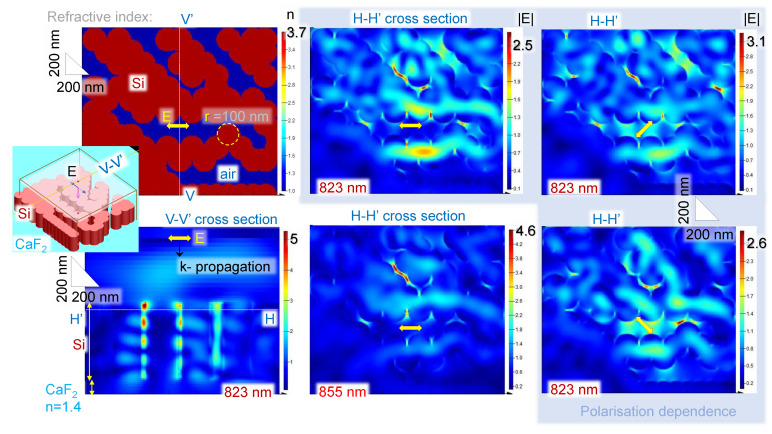
A numerical toy-model of light scattering and enhancement by an Si columnar pattern on CaF_2_ for different orientations of incident linearly polarised *E*-field (0; ±π/4) of light calculated by a finite difference time domain (FDTD) solver (Lumerical). Transverse *V’-V* and longitudinal *H’-H* cross sections show $\left(E/E_{0}\right)$ field enhancement for the incident plane wave $E_{0}=1$. Height of r=100 nm Si cylinders was 0.5 μm; perfectly matching boundary conditions were used. Inset shows 3D rendering of the calculation volume. Permittivity of Si at the selected wavelength was used from the Palik’s database in Lumerical.

In order to qualitatively explain the observed $\cos^{2}\theta$ dependence of the main absorption band at ∼1100 cm${}^{-1}$ (λ=9.09μm in wavelength), a numerical simulation of light field enhancement inside random pattern of sub-wavelengths crevices was carried out for Si nano-cyllinders on CaF_2_. For simplicity, the finite difference time domain (FDTD) calculations were carried at several wavelengths around 0.9 μm, which are shorter by a factor of 10 as compared to the IR wavelengths used. With the same dielectric properties of structures (n(Si)=3.7, $n(\mathrm{Ca}F_{2})=1.4$), a qualitative understanding can be obtained for light enhancement. Figure 6 shows summary of the results of E-field enhancement just 50 nm below the surface of Si cylinders, which are all the same height. The diameter of cylinders was 2r=200 nm and do not correspond to the columnar structures, but were used to form the pattern of the Si layer with different opening sizes (see the refractive index cross section in Figure 6).

**Figure A2:**
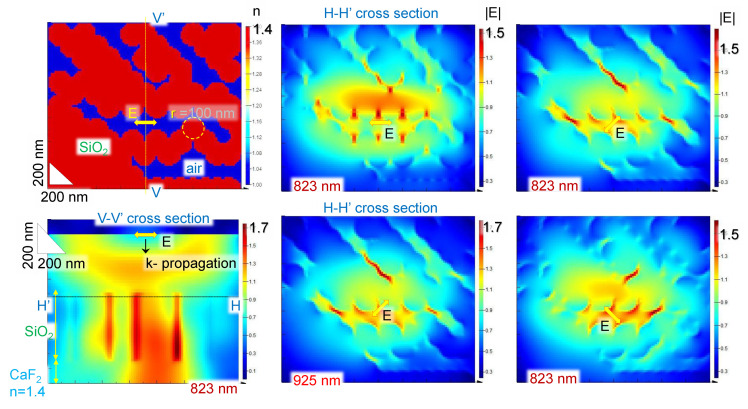
A numerical toy-model of light scattering and enhancement by a SiO_2_ (n=1.4) columnar pattern on CaF_2_ for different orientations of the incident linearly polarised *E*-field (0; ±π/4) of light calculated by finite difference time domain (FDTD) solver (Lumerical). Transverse *V’-V* and longitudinal *H’-H* cross sections show $\left(E/E_{0}\right)$-field enhancement for the incident plane wave $E_{0}=1$. The radius of SiO_2_ cylinders r=100 nm; perfectly matching boundary conditions were used. The inset shows 3D rendering of the calculation volume.

The largest E-field enhancements are at the narrowest sub-wavelength grooves, which are λ/100 and for polarisation orientation perpendicular to the nano-groove. The latter is related to the boundary condition for the E-field normal at the interface between two materials, i.e., $\varepsilon_{Si}E_{Si}=\varepsilon_{air}E_{air}$, where the permittivity is defined by the complex refractive index $\varepsilon ={\tilde{n}}^{2}$. The intensity in the air gap is $I_{gap}\equiv {\left|E_{air}\right|}^{2}=n_{Si}^{4}E_{Si}^{2}$. This scaling qualitatively explains augmented intensity inside sub-wavelength gaps. It is worth noting that the field enhancement exists inside Si as well since the skin depth of evanescent field protrudes up to λ/4 into the volume below the interface. In addition, there is always a strong wavelength dependence as illustrated by two panels for 823 nm and 855 nm wavelengths. For wider nano-gaps λ/10, there is almost not any E-field enhancement (central horizontal gap in panels of Figure 6). For the columnar SiO_2_-on-CaF_2_ sample of the same geometry as Si-on-CaF_2_ (Figure 5b), FDTD simulations predict a weaker enhancement and its localisation as shown in Supplement Figure A2. However, the same orientational dependence of enhancement takes place, i.e., it is the strongest for the normal orientation of the field to the interface.

The qualitative description of E-field enhancement inside sub-wavelength λ/100 grooves is valid for ∼10 μm IR light at which the angular dependence of absorption band was measured (Figure 2b,d). The crevices in the columnar Si-on-CaF_2_ sample (Figure 5) are ∼100 nm long and correspond to the λ/100 at the λ=10μm ($\tilde{\nu }=$1000 cm${}^{-1}$). Once such groove is aligned with the linearly polarised incident synchrotron-IR radiation, field enhancement inside the groove and its vicinity will occur. This enhancement is related to the stronger absorbance and naturally follows the same $\cos^{2}\theta$ angular dependence as observed in FFT maps of SEM images of the surface of columnar Si films.

## 4. Conclusions and Outlook

3D columnar form-birefringent (in visible spectral range λ=0.4–0.8μm) Si micro-films were characterised in the IR fingerprint spectral region 3–10 μm using synchrotron-IR microspectroscopy at Australian Synchrotron IRM beamline. Measurements were carried out from focal volumes, which are sub-wavelength in thickness and with spot sizes 1-to-5 wavelengths in cross section laterally. It is shown that detailed polarisation analysis can be carried out from such small volumes comparable to the wavelength in the IR range. The known material IR absorption bands, e.g., Si-O-Si in Si and SiO_2_, are shown to obey the $\cos^{2}\theta$ orientation dependence as determined by the 4-angle polarisation method. The very same dependence is observed in patterns of nano-crevices on the surface of the films as revealed by Fourier image analysis. By qualitative numerical modeling of light E-field enhancement, it is suggested that the orientational dependence of absorbance follows enhancement (hence stronger absorption) in sub-wavelength λ/100 crevices observed on the columnar Si films. The universal character of E-field enhancement by λ/100 gaps shown by numerical simulations is relevant for qualitative discussion of the observed orientational dependence of absorbance $A\left(\theta \right)\propto \cos^{2}\theta$. Studies of GLAD film formation over laser polymerised, nano-textured and laser ablated patterns [31,32,33] are planned to explore their structure and anisotropy.

## 5. Experimental: Samples and Procedures

### 5.1. Samples

Samples for this study were made using electron beam evaporation technology equipped with two stepper motors for GLAD. Two sets of evaporation have been performed in order to form anisotropic silicon and silica thin films.

For Si coating, polycrystalline wafers of silicon was broken, melted and used as a source of evaporation. The deposition rate was constantly maintained at 3 Å/s and controlled using a crystal quartz monitor. Silicon thin films were coated using a VERA 1100 (VTD, Dresden, Germany) deposition plant.

For silica thin films, granules of SiO${}_{2}$ were evaporated at the same 3 Å/s constant deposition rate. Silica thin films were coated using a SIDRABE (SIDRABE, Riga, Latvia) deposition plant.

In both cases, the thickness of thin films were 1μm, and the coatings were evaporated using the Serial Bi-Deposition technique [34]. During SBD, the substrates were tilted at $70^{\circ }$ angle between its normal and the vapour flux. At the same time, half-turn rotations were performed every six seconds in order to enhance the birefringence of the films and gain better thickness uniformity as well. CaF${}_{2}$ and 〈100〉 orientation p-type Boron doped Si wafers were used as the substrates in both depositions.

Samples were structurally characterised by scanning electron microscopy (SEM) using field-emission SEM mode of Raith 150-TWO (Raith, Dortmund, Germany) electron beam lithography setup. Image processing was made by freeware ImageJ.

### 5.2. Polarised Synchrotron-IR Microspectroscopy in Transmission Mode

The Australian Synchrotron commenced operations for researchers in 2007, with IRM and THz beamlines included in the beamline suite. The details of the synchrotron-IR beam extraction and beamline configuration were previously published elsewhere [35]. The synchrotron-IR experiment was performed on the IRM beamline at the Australian synchrotron (Victoria, Australia), using a Bruker Vertex 80v spectrometer coupled with a Hyperion 3000 FTIR microscope and a liquid nitrogen-cooled narrow-band mercury cadmium telluride (MCT) detector (Bruker Optik GmbH, Ettlingen, Germany). In transmission mode, the IR microscope was operated with a matching 36${}^{\times }$ IR reflecting objective and condenser (NA=0.50). All the synchrotron-IR spectra were recorded within a spectral range of 3800–700 cm${}^{-1}$ using 4 cm${}^{-1}$ spectral resolution. Blackman–Harris 3-term apodization, Mertz phase correction, and zero-filling factor of 2 were set as default acquisition parameters using OPUS 8.0 software suite (Bruker Optik GmbH, Ettlingen, Germany).

Polarisation of the synchrotron-IR radiation has a combination of linear (horizontal along the slit of the first IR-extraction mirror) and circular polarisations [22]. The two components originate from dipole emission inside the bending magnet and at its edge (at the entrance/exit) [20,21]. The IRM beamline receives a higher proportion of the dipole emission compared to the edge emission, which contributes to a larger portion on the THz beamline.

Polarisation is defined as *x*- and *y*-polarisations in the room frame of reference (Figure 1) along the direction of propagation (*z*-axis) in transmission mode. Data analysis was carried out with OPUS 8.0 software (Bruker Optik GmbH).

## Data Availability

Data can be provided upon a reasonable request.

## Appendix A

Light field enhancement calculations by FDTD for SiO_2_-on-CaF_2_ are shown in Figure A2 (it is the same geometry as for Si case shown in Figure 6.)

## References

[1] Taschuk M.T., Hawkeye M.M., Brett M.J. (2010). Glancing Angle Deposition in Handbook of Deposition Technologies for Films and Coatings.

[2] Motohiro T., Taga Y. (1989). Thin film retardation plate by oblique deposition. Appl. Opt..

[3] Grinevičiūtė L., Andrulevičius M., Melninkaitis A., Buzelis R., Selskis A., Lazauskas A., Tolenis T. (2017). Highly Resistant Zero-Order Waveplates Based on All-Silica Multilayer Coatings. Phys. Status Solidi A.

[4] Grinevičiūtė L., Ramalis L., Buzelis R., Tolenis T. (2021). Highly resistant all-silica polarizing coatings for normal incidence applications. Opt. Lett..

[5] Robbie K., Brett M.J., Lakhtakia A. (1995). First thin film realization of a helicoidal bianisotropic medium. J. Vac. Sci. Technol. Vac. Surf. Films.

[6] Grineviciute L., Badorreck H., Jensen L., Ristau D., Jupé M., Selskis A., Tolenis T. (2021). Impact of deposition conditions on nanostructured anisotropic silica thin films in multilayer interference coatings. Appl. Surf. Sci..

[7] Tolenis T., Grinevičiūtė L., Smalakys L., Ščiuka M., Drazdys R., Mažulė L., Buzelis R., Melninkaitis A. (2017). Next, generation highly resistant mirrors featuring all-silica layers. Sci. Rep..

[8] Tolenis T., Grinevičiūtė L., Buzelis R., Smalakys L., Pupka E., Melnikas S., Selskis A., Drazdys R., Melninkaitis A. (2017). Sculptured anti-reflection coatings for high power lasers. Opt. Mater. Express.

[9] Xi J.Q., Schubert M.F., Kim J.K., Schubert E.F., Chen M., Lin S.Y., Liu W., Smart J.A. (2007). Optical thin-film materials with low refractive index for broadband elimination of Fresnel reflection. Nat. Photonics.

[10] Grigoriev F.V., Sulimov V.B., Tikhonravov A.V. (2018). Glancing angle deposition of optical coatings: Results of the full-atomistic simulation. J. Phys. Conf. Ser..

[11] Badorreck H., Steinecke M., Jensen L., Ristau D., Jupé M., Müller J., Tonneau R., Moskovkin P., Lucas S., Pflug A. (2019). Correlation of structural and optical properties using virtual materials analysis. Opt. Express.

[12] Oliva-Ramirez M., Barranco A., Löffler M., Yubero F., González-Elipe A.R. (2016). Optofluidic Modulation of Self-Associated Nanostructural Units Forming Planar Bragg Microcavities. ACS Nano.

[13] MacNally S., Smith C., Spaulding J., Foster J., Oliver J.B. (2020). Glancing-angle-deposited silica films for ultraviolet wave plates. Appl. Opt..

[14] García-Valenzuela A., Alvarez R., Espinós J.P., Rico V., Gil-Rostra J., Palmero A., González-Elipe A.R. (2019). SiO_x_ by magnetron sputtered revisited: Tailoring the photonic properties of multilayers. Appl. Surf. Sci..

[15] Beydaghyan G., Kaminska K., Brown T., Robbie K. (2004). Enhanced birefringence in vacuum evaporated silicon thin films. Appl. Opt..

[16] Doucet A., Beydaghyan G., Ashrit P.V., Bisson J.F. (2015). Compact linearly polarized ceramic laser made with anisotropic nanostructured thin films. Appl. Opt..

[17] Balcytis A., Tolenis T., Wang X., Seniutinas G., Drazdys R., Stoddart P.R., Juodkazis S. (2016). Percolation threshold gold films on columnar coatings: Characterisation for SERS applications. Asian J. Phys..

[18] Ryu M., Honda R., Balcytis A., Vongsvivut J., Tobin M.J., Juodkazis S., Morikawa J. (2019). Hyperspectral mapping of anisotropy. Nanoscale Horiz..

[19] Marshall A.G. (1988). Dispersion vs. absorption (DISPA): A magic circle for spectroscopic line shape analysis. Chemom. Intell. Lab. Syst..

[20] Chao A.W., Mess K.H., Tigner M., Zimmermann F. (2013). Handbook of Accelerator Physics and Engineering.

[21] Chubar O., Smolyakov N. Generation of intensive long-wavelength edge radiation in high-energy electron storage rings. Proceedings of the International Conference on Particle Accelerators.

[22] Ryu M., Linklater D., Hart W., Balcytis A., Skliutas E., Malinauskas M., Appadoo D., Tan Y., Ivanova E.P., Morikawa J. (2018). 3D printed polarizing grids for IR-THz synchrotron radiation. J. Opt..

[23] Ryu M., Ng S., Anand V., Lundgaard S., Hu J., Katkus T., Appadoo D., Vilagosh Z., Wood A., Juodkazis S. (2021). Attenuated Total Reflection at THz Wavelengths: Prospective Use of Total Internal Reflection and Polariscopy. Appl. Sci..

[24] Jutarosaga T., Jeoung J.S., Seraphin S. (2005). Infrared spectroscopy of Si–O bonding in low-dose low-energy separation by implanted oxygen materials. Thin Solid Films.

[25] Alekseev S.A., Zaitsev V.N., Botsoa J., Barbier D. (2007). Fourier Transform Infrared Spectroscopy and Temperature-Programmed Desorption Mass Spectrometry Study of Surface Chemistry of Porous 6H-SiC. Chem. Mater..

[26] Herynková K., Podkorytov E., Šlechta M., Cibulka O., Leitner J., Pelant I. (2014). Colloidal solutions of luminescent porous silicon clusters with different cluster sizes. Nanoscale Res. Lett..

[27] Spizzirri P., Fang J., Rubanov S., Gauja E., Prawer S. (2008). Nano-Raman spectroscopy of silicon surfaces. Mater. Forum.

[28] Juodkazis K., Juodkazytė J., Šebeka B., Savickaja I., Juodkazis S. (2013). Photoelectrochemistry of silicon in HF solution. J. Solid State Electrochem..

[29] Hirschfeld T., Mantz A.W. (1976). Elimination of Thin Film Infrared Channel Spectra in Fourier Transform Infrared Spectroscopy. Appl. Spectrosc..

[30] Han S.M., Aydil E.S. (1997). Detection of combinative infrared absorption bands in thin silicon dioxide films. Appl. Phys. Lett..

[31] Žukauskas A., Malinauskas M., Kadys A., Gervinskas G., Seniutinas G., Kandasamy S., Juodkazis S. (2013). Black silicon: Substrate for laser 3D micro/nano-polymerization. Opt. Express.

[32] Brasselet E., Gervinskas G., Seniutinas G., Juodkazis S. (2013). Topological shaping of light by closed-path nanoslits. Phys. Rev. Lett..

[33] Buividas R., Rekštytė S., Malinauskas M., Juodkazis S. (2013). Nano-groove and 3D fabrication by controlled avalanche using femtosecond laser pulses. Opt. Mat. Express.

[34] Hodgkinson I., Hong Wu Q. (1999). Serial bideposition of anisotropic thin films with enhanced linear birefringence. Appl. Opt..

[35] Cheeseman S., Khanh Truong V., Vongsvivut J., Tobin M.J., Crawford R., Ivanova E.P. (2019). Applications of Synchrotron-Source IR Spectroscopy for the Investigation of Insect Wings.

